# ‘We Don't Have to Prove to People How We're Feeling’: Understanding the Role of Peer Support Groups in Countering Epistemic Injustices in Long COVID at a US Centre

**DOI:** 10.1111/hex.70266

**Published:** 2025-04-12

**Authors:** Nandini Sarma, Sam Gage, Catherine L. Hough, Aluko A. Hope

**Affiliations:** ^1^ Department of Medicine University of California Davis School of Medicine, Division of Pulmonary, Critical Care, and Sleep Medicine Sacramento California USA; ^2^ Department of Medicine Division of Pulmonary, Allergy and Critical Care Medicine Oregon Health & Science University Portland Oregon USA

**Keywords:** epistemic injustice, Long COVID, peer support, qualitative study

## Abstract

**Background:**

Long COVID, an infection‐associated chronic condition characterised by new or worsening signs or symptoms for more than 3 months after a SARS‐CoV‐2 infection, is a chronic debilitating illness which remains poorly understood. Epistemic injustice in healthcare describes the unique harms or wrongs done to a person in their capacity to share and acquire knowledge about their illness. Although the concept of epistemic injustice has been described in other chronic conditions, few studies have explored these concepts in Long COVID.

**Objectives:**

(1) To understand the lived experience of epistemic injustice in adults with Long COVID who were participating in a peer support group intervention and (2) to describe the potential impact of the support group on these experiences in participants.

**Methods:**

Qualitative analysis utilising inductive analysis of semi‐structured individual interviews of patients with Long COVID who participated in a peer support group intervention at an academic medical centre in Oregon, USA.

**Results:**

We identified three themes that captured the lived experiences of epistemic injustice in Long COVID support group participants: (1) dismissal and disregard; (2) episodic and unpredictable symptoms and impairment, and (3) knowledge and interpretation practices. We also found that the peer support potentially impacted these experiences of epistemic injustice through (1) recognition and validation; (2) solidarity and community, and (3) information exchange and expectation setting.

**Conclusions:**

Long COVID patients are at risk of experiencing epistemic injustice in seeking healthcare for this complex condition. Peer support programmes may be one approach to help counter these experiences and should be further studied as a complex intervention for improving patient‐centred care in Long COVID.

## 1 Introduction

Since the early use of the term by patients to capture their lingering symptoms after acute COVID‐19, Long COVID has become the most widely used term to capture the chronic conditions that can occur after SARS‐CoV‐2 infection [1]. Although no universally accepted definition exists, the scientific community increasingly recognises Long COVID as one of several infection‐associated chronic conditions, characterised by persistent symptoms affecting one or more organ systems [2, 3, 4]. Most recent estimates from the CDC suggest that around 6.9% of US adults may have had Long COVID and that approximately 14 million adults may have been impacted by this condition [5]. Long COVID has a long‐term impact on patients' health and function and has been described as a ‘mass disabling event’ for the 21st century [6, 7, 8, 9, 10, 11].

Although the medical and scientific understanding of Long COVID has significantly advanced since the pandemic began, challenges remain. The absence of a specific objective biomarker, the complex interplay of biopsychosocial factors affecting patients' health and well‐being and the lack of evidence‐based pharmacologic treatments continue to create substantial hurdles [12, 13, 14].

Epistemic injustice, initially described by the philosopher Mirander Fricker, is a form of injustice to a person in their capacity as a knower, interpreter or provider of information and knowledge [15, 16]. There is a growing body of theoretical research on how epistemic injustice may be relevant to the quality of healthcare that patients are able to receive for certain chronic conditions [17, 18, 19]. At the individual level, epistemic injustice may include such things as patients being ignored, dismissed or silenced or might also include clinicians' negative prejudices about certain categories of patients [18, 19, 20]. At the level of the health system, epistemic injustice may intersect with other axes of oppression, including gaps in the scientific understanding of the condition or limitations in the biomedical model that complicate patients' ability to be diagnosed and treated [15, 16, 18, 19, 21, 22, 23, 24, 25].

Although there have been several recent empirical studies that have captured examples of epistemic injustice in Long COVID care such as stigma, mistrust and invisibility, and the experiences of patients during the prolonged ‘diagnostic odysseys’ of Long COVID diagnosis, this area of inquiry is still largely underexplored [26, 27, 28]. Management guidelines for Long COVID recommend that patients receive advice and information on self‐management, including through support groups and other forms of psychosocial support [29]. Peer support groups have been previously studied in other chronic conditions as an intervention that can leverage the strengths and experiential knowledge from a group of patients to improve their well‐being [30, 31, 32, 33]. Various types of support groups have been studied in Long COVID as a result of these management guidelines [34, 35]. The stigma surrounding Long COVID and the reliance on mental health practitioners to facilitate peer support groups highlight a critical need to understand how support groups can potentially counteract epistemic injustices in Long COVID care [36, 37]. Therefore, we sought to understand the lived experiences of epistemic injustice in support group participants and the potential impact of the peer support intervention on these injustices.

## 2 Methods

### 2.1 Study Design and Setting

Healthy Outcomes for Long COVID through Peer Support (HELP) was an 8‐week facilitated pilot support group intervention designed for adult patients being treated for Long COVID at a tertiary care centre in Portland, Oregon, USA. The support groups were designed based on an outpatient model of peer support in which the role of the clinicians is to help create a safe space for patients to take and give support to each other with mutual respect [32, 38]. This is an analysis of qualitative data from individual semi‐structured interviews that was aimed at understanding the safety, effectiveness and implementation barriers of the peer support intervention within a tertiary care Long COVID programme.

Long COVID patients could be invited into the programme if they were (1) able to participate in an intake conversation with the support group staff and expressed understanding of the ground rules and expectations of the programme; (2) were deemed safe for participation by the multidisciplinary Long COVID clinical team managing the patient based on their level of symptoms and the stage of their recovery, and (3) were comfortable conversing in English. The first support group occurred from October through December 2021 and was facilitated by a staff social worker; the second support group occurred from October through November 2022 and was facilitated by psychiatry postgraduate trainees with supervision from an experienced social worker with experience leading cancer support groups. This study is reported using the Consolidated Criteria for Reporting Qualitative Data (COREQ) checklist and was approved by the university's Institutional Review Board (#00023311).

### 2.2 Participant Selection

Upon completion of the HELP programme, all support group participants were invited via phone to participate in a qualitative evaluation. All participants who consented were interviewed. There were no additional exclusion criteria. Each participant was reimbursed $10 for completion of the interview in recognition of the effort and time utilised while having a chronic and disabling condition. Participants were fully informed about the study's purpose and how their data would be used and stored. Verbal consent was obtained before the interview, as the study was deemed to pose minimal risk, with no more discomfort than routine activities. The date and time of consent were documented instead of a signature. At the start of each recording, participants confirmed their name, agreement to participate and the current date.

### 2.3 Data Collection

Three of the investigators (A.A.H., S.G. and C.L.H.) were involved in drafting the interview guide, which focused on categories such as motivations for participation, impact on recovery, benefits, challenges and potential harms of participation, lifestyle changes and overall experiences (Appendix A). These categories were selected by the investigators a priori based on current literature regarding peer support in critical and acute illness recovery [34, 39, 40, 41]. One investigator (S.G.) led the training of the research staff who conducted the semi‐structured interviews in English via phone or secure video conferencing platform. The interviews occurred between 10 December 2021 and 30 June 2022. Interview recordings were de‐identified and anonymised via assignment of a unique study ID by the interviewer. Interviews were recorded and transcribed verbatim by trained staff and reviewed for transcription accuracy and consistency by a second interviewer.

### 2.4 Data Analysis

During the primary analysis of the qualitative data which focused on understanding the potential effectiveness of the pilot support group intervention, A.A.H. and S.G. had familiarised themselves with the data, created transcript summaries iteratively and developed a codebook by thematic identification and categorisations based on the domains of the interview guide and a conceptual framework of peer support [32]. For this analysis, we used an inductive thematic analytic approach since this allowed for the greatest flexibility and because there was no previously validated framework for epistemic injustice in healthcare [42, 43]. A.A.H. and N.S. reviewed the interview transcripts to familiarise themselves and build consensus on ways in which participants described their lived experiences of epistemic injustice in the transcripts. A.A.H. and N.S. created a preliminary codebook and then sequentially coded three interview transcripts at a time. After each sequence, the codebook was revised for consensus. We used ATLAS.ti (version 22) to organise data, code transcripts and track decisions related to analyses. Data saturation was determined when no new codes or themes were observed across the interviews. The analytic team (A.A.H., S.G. and N.S.) shared key bibliographic references, code reports and summaries to ensure ongoing agreement on the findings. Preliminary codes were merged into themes, and the names of themes were revised iteratively, including again during the reporting and writing phase of the project. Importantly, in finalising the names of themes capturing the impact of the support group on epistemic injustice, we aimed to have the names encompass both the potential for epistemic harm and benefit. Key quotes were extracted for reporting by A.A.H. and S.G.

### 2.5 Reflexivity and Positionality of the Research Team

The group of investigators for these analyses included a long COVID survivor that was treated at the Long COVID programme but did not participate in the support group (S.G.), a medical director of the Long COVID clinical programme (A.A.H.) and two investigators not directly involved in the Long COVID programme (N.S. and C.L.H.). The interdisciplinary nature of this group of investigators likely contributed to the validity of the analysis through triangulation. Three coders (S.G., A.A.H. and N.S.) provided a level of intersubjectivity, rigour and scrutiny to the coding process.

## 3 Results

Of the 22 participants in the peer support programme, 16 consented to participate in our qualitative assessment. In total, fourteen participants identified as female, two identified as Hispanic and fifteen identified as White (Table 1). The median number of sessions attended was 6 for Group 1 and 5.5 for Group 2. In qualitative analyses, we found that three themes captured the lived experiences of epistemic injustice that were described by participants. We also found that three themes captured the potential impact of the peer support intervention on these epistemic challenges. Extended quotes exemplifying each theme are presented in Tables 2 and 3.

**Table 1:** Participant characteristics.

Patient 3	44‐Year‐old White female whose first SARS‐CoV‐2 infection occurred in August 2020
Patient 5	76‐Year‐old White and American Indian/Alaska Native female whose first SARS‐CoV‐2 infection occurred in March 2020
Patient 6	74‐Year‐old White female whose first SARS‐CoV‐2 infection occurred in March 2020
Patient 7	51‐Year‐old White female whose first SARS‐CoV‐2 infection occurred in April 2020
Patient 8	59‐Year‐old White female whose first SARS‐CoV‐2 infection occurred in March 2020
Patient 9	55‐Year‐old White female whose first SARS‐CoV‐2 infection occurred in May 2020
Patient 10	54‐Year‐old White female whose first SARS‐CoV‐2 infection occurred in March 2020
Patient 11	35‐Year‐old White female whose first SARS‐CoV‐2 infection occurred in August 2020
Patient 12	65‐Year‐old White female whose first SARS‐CoV‐2 infection occurred in March 2020
Patient 13	44‐Year‐old White and Hispanic female whose first SARS‐CoV‐2 infection occurred in April 2020
Patient 14	55‐Year‐old White female whose first SARS‐CoV‐2 infection occurred in December 2020
Patient 15	67‐Year‐old White female whose first SARS‐CoV‐2 infection occurred in March 2020
Patient 16	34‐Year‐old White and Hispanic female whose first SARS‐CoV‐2 infection occurred in March 2020
Patient 17	26‐Year‐old White individual whose first SARS‐CoV‐2 infection occurred in December 2020
Patient 18	44‐Year‐old female whose first SARS‐CoV‐2 infection occurred in March 2020
Patient 20	52‐Year‐old White male whose first SARS‐CoV‐2 infection occurred in April 2020

**Table 2:** Themes of epistemic injustice in lived experiences of patients with Long COVID.

	Dismissal and disregard	Episodic symptoms and impairment	Knowledge practices and interpretation
Individual	‘I feel bad whining…when there's so many others that have died or…have been impacted more severely. I didn't want to take services or time away from those that are in the ICU or bad shape in the hospital’ (P14)	‘I'm having such a hard time even showing up for me…that is the hard part, is …potential over‐exhaustion’ (P8) ‘I have noticed, and I don't know if there's a place for this with your questions today, but I feel like it's important to note. Something very weird has been happening where literally about every two months I am sure I have COVID again, and I have symptoms, I have mild fever, I have chills, I ache all over, I have a headache, I have a little bit of a dry cough, and I'm sure I have COVID. I test negative on a rapid test, I test negative on a PCR.’ (P15) ‘Exercise and activity have been difficult because of the post‐exertional fatigue, but also the untreated chronic and acute pain I've been in. I feel like I'm [age] years old in an 85‐year‐old body.’ (P14)	‘It seems like every new symptom is new to me, and its new to the doctor and its like youre lost…nobody can help you. And you don't know whats going on with you. And when your doctor doesn't know what's going on with you, then what do you do?’ (P18) ‘I was looking for patterns—does this flare‐up happen after I do too much? Is it related to food? Am I getting re‐infected? I started keeping a log of everything because no one had answers for me.’ (P15) ‘Some of the symptoms are so similar to fibromyalgia symptoms that I don't know if they went away, or if they just sort of blended into what was already there.’ (P5)
Interpersonal	‘People all the time, they look at me like, “oh, you look great” and I'm like, it doesn't mean that I feel great’ (P13)	‘One day I can walk and talk and the next day I can't and I can't get out of bed and they don't understand that. And I don't fully understand that’ (P16)	‘My family has really struggled to understand what I'm going through … we really need a family education component right away.’ (P16)
Health system	‘So many, almost all of us, at one point or another, have had the door shut in or face, or told, you know, no, that's not what it is and we just have to go home and suffer more’ (P18) ‘I have never been so gaslight in my entire life consistently day in and day out…“I fought hard because the [specialist name] didn't believe that COVID was a real thing, and so I had to fight multiple appeals to get to see a [specialist name] that did.”’ (P8) ‘Yeah, this [Long COVID program access] is part of my frustration‐ is that I only had one appointment, and I basically have tried to get into more and I've been told I can't or its not possible’ (P7)	‘My energy levels and my fatigue and brain fog tend to be worse, so there were times where I would have liked to participate more but felt like I was just too fatigued to be able to say a whole lot’ (P10) ‘The first year of my illness, I couldn't even look at my email. So… with the support group, one big thing for me is, it [the invitation] wasn't showing up in my MyChart. … they were doing everything via personal email, but … I couldn't start using email until really a couple of months ago.’ (P16)	‘We, at this point, know more than they do, because we live with this every day and as you can tell, I…barely have enough energy to comment a little on it, so…I think the more that they learn the more they will help others…. And I know this will change. It was scary for several of us that were talking together to see how little is known by the specialists’ (P3) ‘It was interesting to me that so many people had the same experience with doctors that I had been having because nobody knew what was going on. And a year and a half into the COVID pandemic, they still didn't know, and I don't get that. It seems to me, like, if you're a doctor and you're treating people who are saying that they've got these symptoms ever since they had COVID, that you would start reading up on Long COVID’ (P5)

**Table 3:** Themes encompassing the impact of the support group intervention.

	Recognition and validation	Solidarity and community	Information exchange and expectation setting
Positive experiences	‘We were heard. We …supported each other and we felt validated because we were heard…. Like its been just so hard on top of being sick, that just to have a nice place with people that understand that you just slept, like 20 out of 24 hours and you haven't combed your hair in 2 days…like it just validates you that…this is normal for where I'm at in this illness.’ (P18)	‘Just being able to reach out after the meetings…its so nice…you don't feel like you're the Lone Ranger.’ (P12) ‘I think the most number one most effective aspect is just being in the community and knowing that you're not alone.’ (P10)	‘Gathering information and being given …new information about where to seek help at, and additional resources for recovery and support too, has been really good.’ (P12) ‘It gives me basic place of understanding and advocacy for me to take forward to my doctor, to my friends to help understand and into my greater community.’ (P8) ‘The biggest thing for me was knowing that people are investigating [Long COVID] in the first place.’ (P17)
Potential negative experiences	‘I have a pretty strong “pick yourself up by your bootstraps” kind of attitude that works for me …I think when I listened to other people talking about all of the things that are bothering them, instead of making me feel stronger or supporting them, it just debilitates me.’ (P15) ‘Ours is a very different experience from the people who were totally healthy and then just got brought down by COVID and they're now living our lives basically for the first time, and they are devastated. And they're like, but I still have hope, and were 20 years in going, good luck with that.’ (P03)	‘We had different backgrounds, of course and different stressors that were going on and different symptoms.’ (P20) ‘I wasn't comfortable sharing a lot with the group through the zoom type format…the group was so large that we really didn't get a chance to interact so that we could get comfortable with each other.’ (P5)	‘He [facilitator] made some suggestion when someone was talking about their symptoms…that it would be helpful for people to meet with their Long COVID provider pretty regularly…and I thought, oh, I got one appointment. And so from that, I reached out to the Long COVID clinic and the provider …but I was told that she wasn't going to be seeing people in person until the new year.’ (P07)

### 3.1 Lived Experiences of Epistemic Injustice

#### 3.1.1 Dismissal and Disregard

This theme encompassed experiences of invalidation, neglect and being disbelieved at multiple levels: intra‐personally, as patients grappled with self‐doubt; interpersonally, as they faced a lack of recognition or validation from family and friends, and within the healthcare system, where they encountered scepticism, misdiagnosis and medical dismissal from professionals. One participant described the multiple levels of dismissal and disregard in this way:

I look just fine, you know, people all the time, they look at me like, ‘oh, you look great’ and I'm like, it doesn't mean that I feel great…. We had a talk [in the support group] about‐ we don't have to prove to people how you're feeling, and this is real, right‐ this is really happening? Because people question, you know, people see you looking just fine. This makes you question your sanity, like am I really going through this? Why am I really this tired? Right? Like, am I really not being able to read right now? You just question yourself.

(P13)

The dismissal and disregard often increased the effort required to navigate the healthcare system as reflected in these comments:

So many, almost all of us, at one point or another, have been‐ had the door shut in our face or told, you know, no, that's not what it is, and we just go home and suffer some more…. all the literature that I found, I found on my own. And I've educated…my doctors…[it's] unbelievable that I'm educating my doctor.

(P18).

I fought hard because the [specialist name] didn't believe that COVID was a real thing, and so I had to fight multiple appeals to get to see a [specialist name] that did.

(P8)

#### 3.1.2 Episodic and Unpredictable Symptoms and Impairment

The nature of the symptoms and impairments that patients experience, particularly their episodic and unpredictable nature, contributed to feelings of frustration and social isolation. The fluctuating nature of the symptoms often created a discordance between participants' desire to engage in the support group and their capacity to do so. One participant described the interpersonal impact of the fluctuating impairment this way:

My family still doesn't understand it because one day I can walk and talk, and the next day I can't, and I can't get out of bed, and they don't understand that. And I don't fully understand that.

(P16)

Another participant described how the fluctuating symptoms impacted their capacity to engage in the support group intervention this way:

At [the time of day of the group] my energy levels and my fatigue and brain fog tend to be worse, so there were times where I would have liked to participate more but felt like I was just too fatigued to be able to say a whole lot.

(P10)

#### 3.1.3 Knowledge and Interpretation Practices

Participants frequently talked about how the lack of scientific knowledge about the illness shaped their experiences with Long COVID. This theme encompassed the active processes by which individuals sought to understand their condition, including asking questions, conducting personal research, analysing and interpreting symptoms, evaluating medical evidence and engaging in discussions about different perspectives on the illness. Given the evolving and uncertain nature of Long COVID, participants often found themselves in a continuous cycle of information‐seeking, whether through medical consultations, peer discussions or independent investigation. One participant described the intra‐personal effect this way:

It seems like every new symptom is new to me, and it's new to the doctor and…it's like you're lost…nobody can help you. And you don't know what's going on with you. And when your doctor doesn't know what's going on with you, then what do you do?

(P18)

For many, the lack of clear medical consensus led to critical thinking and self‐advocacy, as they had to navigate conflicting advice, question the credibility of information sources and make informed decisions about their care. Some participants expressed frustration with the medical system's knowledge gaps, whereas others described debating competing interpretations of symptoms—both within themselves and with healthcare providers. For example, one participant described the challenges in differentiating between pre‐existing medical conditions and Long COVID symptoms this way:

My question is, you know, look at these anxiety symptoms and look at these dysautonomia symptoms. There's all this crossover. How do you know anyway?

(P9)

This theme encompassed how participants understood their own illness and how medical professionals communicated about their level of knowledge or uncertainty regarding their condition. One participant shared appreciation for honesty from medical providers in this way:

When I have providers who say to me, ‘I don't know if you'll ever get better,’ you know, in some ways I feel like, at least they're being real with me in telling me that they don't know. It's the doctors that don't know and don't have an answer and then do this gaslighting situation or avoidance that causes much more harm.

(P7)

Another participant reflected on the importance of humility among the healthcare providers they encountered in the following:

It was really important and nice that…[the practitioner] expressed so humbly that…all answers are okay, that, you know, we don't have answers.

(P8)

### 3.2 The Impact of Peer Support on Epistemic Injustices

#### 3.2.1 Recognition and Validation

Participation in the peer support group offered participants a space in which their illness and struggles were acknowledged, understood and validated. Recognition was described in multiple ways—from hearing others describe nearly identical struggles, to receiving emotional support from peers, to recognising systemic failures in healthcare. The act of being believed and understood helped combat the isolation many had felt when their symptoms were questioned or minimised. As one participant described:

…just to have a nice place with people that understand that you just slept, like, 20 out of 24 hours and you haven't combed your hair in 2 days. Maybe 4 days instead of 2… Like, it just validates you that…this is normal for where I'm at in this illness.

(P18)

Another participant emphasised:

Getting to hear from other people that are living your same experience when your doctors are telling you it doesn't exist is definitely important.

(P3)

Beyond personal recognition, participation in the support group also illuminated structural injustices, as participants realised their struggles were not just personal failings or bad luck but part of a broader pattern of systemic neglect:

It was also helpful for me to realize I'm not the only one who is having a horrific experience at [hospital name]…hearing and seeing it in the faces of other people and their own personal struggles with [hospital name], it's like oh, it's just the frickin' system. They don't really care about anybody.

(P8)

Not all participants described feeling validated by the support group. For some, the act of witnessing others' struggles was potentially emotionally overwhelming or even counterproductive. One participant said:

I have a pretty strong ‘pick yourself up by your bootstraps’ kind of attitude that works for me …I think when I listened to other people talking about all of the things that are bothering them, instead of making me feel stronger or supporting them, it just debilitates me.

(P15)

#### 3.2.2 Solidarity and Community

Most participants emphasised that the peer support group fostered a sense of belonging and community, offering a crucial counterbalance to the isolation and lack of understanding many had faced from medical providers and loved ones. Beyond offering community, the group became a foundation for both personal and collective empowerment, with participants finding meaning in ongoing friendships, structured peer interaction and even collaborative advocacy efforts. One participant reflected on the profound impact of simply being in a shared space with other patients:

I think the most, the number one most effective [aspect] is just being in the community and knowing that you're not alone in what you're feeling or doing…. It was fairly profound…

(P10)

For others, the consistency of the group provided stability, reinforcing a sense of connection over time:

There's power in seeing, just simply seeing the same faces each week… ‘oh, you are a community of people who I'm traveling with, I'm not alone.’

(P8)

However, participants also recognised the delicate balance of engagement, acknowledging that chronic illness often made it difficult to sustain additional relationships. Some appreciated the structured nature of the group, which allowed for meaningful interaction without becoming overwhelming:

I'm not in the space to, like, bring more people in, necessarily, because I can't take care of myself …I want to be more connected with more people, but… I don't want it to drain you…And so I think that's what's nice about a peer group is because it's like a very specific kind of interaction.

(P11)

Although many found comfort in the group setting, a few participants struggled to feel fully integrated due to the format and group size. Some found the virtual environment limiting, making it harder to build trust and engage meaningfully:

I wasn't comfortable sharing a lot with the group through the zoom type format…the group was so large that we really didn't get a chance to interact so that we could get comfortable with each other.

(P5)

#### 3.2.3 Information Exchange and Expectation Setting

Participants described the peer support group as a critical space for information exchange, where they gained valuable resources, provided materials and discussed symptom management, healthcare navigation and self‐advocacy. Hearing the experiences of others provided guidance on where to seek help and what resources were available:

Hearing similar stories, gathering information, and being given information too, new information about where to seek help at, and additional resources for recovery and support too, has been really good.

(P12)

Although medical and symptom‐specific guidance—such as recommendations on antihistamines, sleep aids and lifestyle modifications—was useful, participants emphasised that learning how to interact with and advocate for themselves within the healthcare system was particularly impactful. The group helped them understand their rights as patients and empowered them to push for better care in medical and community settings:

It gives me a basic place of understanding and advocacy for me to take forward to my doctor, to my friends to help understand and into my greater community.

(P8)

Participants described how the knowledge gained in sessions helped them feel more confident in challenging dismissive medical responses and taking proactive steps in their care:

I think in everybody else's recovery, that's what they're coming to—the conclusion is to advocate for yourself, you know, and I kinda think that the group gives that strength to know that…. The information given out at the meetings and stuff is very beneficial to…know where at least to a) to begin or b) to follow up with.

(P12)

A key area of learning was energy management, which many participants connected to changes in the way they related to their fatigue and their overall approach to their condition. The group provided strategies for pacing and boundary‐setting, helping individuals develop a more sustainable mindset around activity and rest:

I think we talked a lot about energy and conserving energy and understanding more of that. So it made me more mindful of that.

(P13)

For some, learning about the energy cycle helped them reframe their experiences and set healthier boundaries, which improved both their personal and professional lives:

I know that they did a really great little kind of explanation of energy… and how we, if we feel good, we're going to overdo it. And then we get in that cycle of…really burnt out to, like, having a little bit of energy and trying to use it. And so… that's one of the things that I would say I made a lifestyle choice on—just like, being more consistent with giving myself the time to rest… So, once I have ways to define the things I'm experiencing, which—the group did help me define the things I was experiencing—I now can set boundaries… and it also helps with my, like, my professional life.

(P17)

At times, however, the support group also revealed gaps in the healthcare system that participants were unaware of, leading to frustration. In one instance, a participant assumed they would receive more follow‐up care based on group discussions, only to realise that their expectations were misaligned with actual medical practices:

He [the facilitator] made some suggestion when someone was talking about their symptoms… that it would be helpful for people to meet with their Long COVID provider pretty regularly… and I thought, oh, I got one appointment. And so from that, I reached out to the Long COVID clinic and the provider …but I was told that she wasn't going to be seeing people in person until the new year.

(P7)

## 4 Discussion

**Figure 1:**
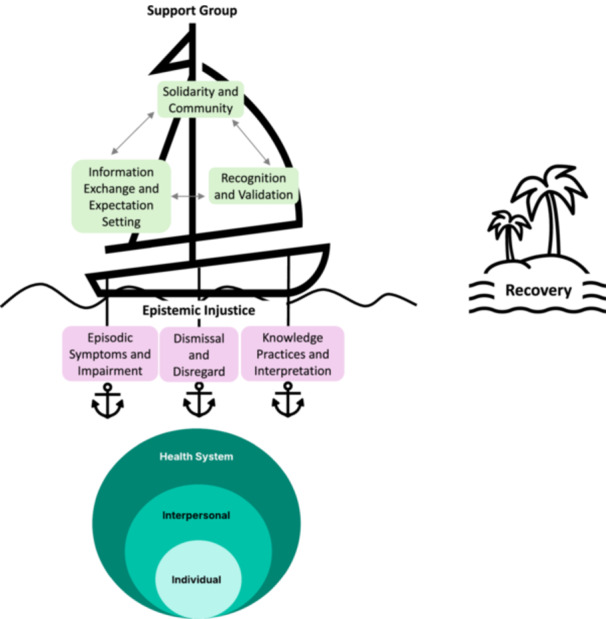
Conceptual model of the role of support groups in countering epistemic injustice experienced by patients with Long COVID.

In our qualitative study of Long COVID patients who participated in a pilot peer support intervention embedded within a US‐based tertiary care Long COVID programme, we found that all participants described ways in which their ability to fully understand their condition or contribute to the health system's understanding of their illness was hindered. Specifically, we identified three broad themes of epistemic injustice in their lived experiences: dismissal and disregard; episodic and unpredictable symptoms and impairments, and knowledge and interpretation practices. These experiences were not confined to individual interactions but spanned multiple levels, including the intra‐personal, interpersonal and health system levels, suggesting that epistemic injustice operates within a broader socioecological framework. Additionally, we identified three themes representing potential mechanisms through which our peer support intervention may have influenced participants' experiences of these injustices. We think these results have implications for how peer support interventions can be leveraged to improve health outcomes in Long COVID and other poorly understood medical conditions. We propose a tentative conceptual model for the role of support groups in addressing the lived experiences of epistemic injustices in Long COVID represented in Figure 1.

The theme of ‘disregard and dismissal’ echoes themes of epistemic injustice that have been described in other chronic conditions including HIV, mental health disorders and chronic fatigue [23, 37, 44, 45]. Our findings show how these experiences undermine patients' recovery by eroding self‐efficacy, increasing emotional distress and weakening the therapeutic alliance between patients and providers. This theme of dismissal and disregard has been previously described in Long COVID patients in both qualitative and quantitative studies [26, 28, 46]. For example, Ireson et al. conducted a qualitative analysis of patient stories from an online COVID‐19 community early in the COVID‐19 pandemic and found that patients reported feeling like their symptoms were not believed by the medical establishment which often led them to seek alternative approaches to managing their illness including through online support groups [26].

The theme that we called ‘knowledge and interpretation practices’ reflects the ways in which our participants became aware that their chronic condition was not well understood by the medical and scientific establishment. Sociologists have previously characterised certain conditions—such as fibromyalgia, endometriosis, chronic pain and chronic fatigue syndrome—as ‘low prestige’ illnesses, similar to Long COVID in that they lack objective diagnostic tests and disease‐modifying treatments [47]. Our findings support the central need for epistemic humility and solidarity in the patient‐centred care revolution [24, 48, 49]. Participants expressed a deep desire for their involvement in the support group to contribute to the broader production of knowledge about Long COVID—making their suffering more visible through a process of co‐production akin to how the term ‘Long COVID’ itself emerged [1].

Our findings regarding the impact of the peer support intervention, specifically regarding themes of validation, community and information exchange, reflect other literature on the role of peer support in improving participant well‐being [30, 31, 32, 33]. We found few evidence that patients in our study were poised for true epistemic resistance, or explicit actions to ‘change oppressive normative structures’, by leveraging their collective lived experience to change or undermine these epistemic structures within healthcare [36]. Rather, our results would suggest that support groups may potentially counter these epistemic injustices by allowing: recognition and validation; solidarity and community; information exchange and expectation setting. These findings are consistent with a relationship‐based approach to care, which has been described in narrative medicine and may allow patients to find meaning in the face of medical uncertainty [50, 51].

We found that the episodic and unpredictable symptoms and impairments that patients experienced as part of having Long COVID contributed to epistemic injustice not only because it impacted how they were able to monitor their illness and how they were perceived by their loved ones or their clinicians but also because they were often less able to accrue the benefit of the support intervention. These results are consistent with the literature on episodic disability, which has previously been characterised in patients with HIV/AIDS where both extrinsic and intrinsic contextual factors impacted participant care experiences [52, 53, 54, 55]. The episodic nature of the symptoms and impairments in Long COVID also has important implications for how complex psychosocial interventions may need to be designed and implemented to be most effective. Across multiple studies, post‐exertional malaise is a symptom that has been well described in Long COVID patients whereby patients' symptoms get worse after physical, cognitive, emotional exertion [56]. Since patients may struggle to access care due to the episodic nature of these symptoms, more research on how to improve health systems to address post‐exertional malaise is needed. Our participants also point to potential strategies that may be useful for tailoring peer support interventions to better acknowledge Long COVID symptoms. These strategies include providing information in multiple formats that can be accessed at any time, being flexible in terms of nature and type of attendance to accommodate for symptoms, focusing on local health systems and the need for trauma‐informed approaches.

The strengths of our study include the sampling of participants from two cycles of our support group intervention and the incorporation of individuals with Long COVID in the development of this study. The weaknesses of our study include single‐centre design, which may limit further exploration into the impact and prevalence of epistemic injustice, the inclusion of only English‐speaking participants and the fact that it was conducted within a large tertiary‐care academic medical centre, which may have influenced participant recruitment and experiences. Lastly, our study participants were predominantly White, middle‐aged to older women, reflecting the demographic of patients treated for Long COVID at our quaternary care clinic during this period. This aligns with broader findings that women are at higher risk of developing Long COVID than men [57, 58]. Although our sampling process did not intentionally exclude men, it remains plausible that the experiences of epistemic injustice observed in our study are shaped in part by gender bias and may not be fully generalisable to male Long COVID patients. Notably, women are more likely to seek medical care for persistent, multisystem conditions such as autoimmune diseases, chronic fatigue syndrome, fibromyalgia and multiple chemical sensitivities—conditions that, like Long COVID, remain poorly understood within a biomedical framework that privileges organ‐specific pathology [59]. Feminist philosophers of science have long critiqued the biomedical field's strong positivist orientation, arguing that it often marginalises the relevance of patients' lived experiences and social contexts in the production of medical knowledge [60]. Given these dynamics, epistemic injustice may be particularly salient for women with Long COVID. Future studies should seek to include a diverse sample across gender and other demographic variables to better understand how epistemic injustice manifests in different populations.

## 5 Conclusion

In this qualitative study of Long COVID patients who participated in a support group intervention, we described the lived experience of epistemic injustices across themes of dismissal and disregard, episodic symptoms and impairment, and knowledge and interpretation practices, that are present at the intra‐personal, interpersonal and health system levels. We also identified potential mechanisms through which support groups can counter these experiences through recognition and validation, solidarity and community, and information exchange and expectation setting. Patient's lived experiences of epistemic injustice support the importance of a relationship‐based approach to medicine and the importance of epistemic humility in patient‐centred care. Our findings highlight participant‐identified structures for how support interventions may be implemented, including flexibility in format, focus on local resources and multiple modes of information sharing. However, how psychosocial support interventions can be better structured to counter epistemic injustice for Long COVID and other chronic illnesses should be the source of further innovation and research.

## Author Contributions

**Nandini Sarma:** writing – original draft, methodology, validation, visualization, writing – review and editing, formal analysis, data curation, resources, conceptualization. **Sam Gage:** conceptualization, investigation, writing – original draft, writing – review and editing, data curation, resources, project administration, software. **Catherine L. Hough:** investigation, supervision, conceptualization, visualization. **Aluko A. Hope:** conceptualization, investigation, funding acquisition, writing – original draft, writing – review and editing, methodology, validation, formal analysis, supervision.

## Disclosure

A preliminary version of this work was presented at the American Thoracic Society International Conference on May 22, 2023, in Washington, DC.

## Ethics Statement

This study was approved under Institutional Review Board # 00023311.

## Consent

Patients with Long COVID who had participated in a pilot peer support intervention were interviewed to better understand the potential effectiveness of the programme and to provide recommendations on how to improve the programme. One investigator (S.G.) also identified as a patient with Long COVID, and another investigator (A.A.H.) was the medical director of the Long COVID Programme at which the pilot peer support intervention was conducted.

## Conflicts of Interest

The authors declare no conflicts of interest.

## Data Availability

The data that support the findings of this study are available on request from the corresponding author. The data are not publicly available due to privacy or ethical restrictions.

## Appendix A Participant Interview Guide

Thank you for agreeing to participate. We are trying to understand your opinions of and experiences with the peer support programme at OHSU. Because of your experience with the programme, we are hopeful that you can help us understand what aspects of the programme could work for future long COVID patients. There are no right answers. The interview will be recorded but is confidential and the transcript will not have any identifiable information about you. Do you have any questions before we get started?

**Pre‐Interview Demographics:**

[]How many sessions of the peer support groups were you able to attend?

[]Level of education

[]Level of interaction with the OHSU programme

[] Age, Gender

Questions

1. Can you share with us your motivations for participating in the peer support programme?
2. How comfortable were you sharing or engaging during the group sessions?
  a. Probe: Did that experience change over time?

3. Can you share a story from your experience in the group that really stands out for you?

4. *[The peer support group was on a virtual platform for 90 min with invited guests and opportunities to share your story*.*]* What specific aspects about the structure and process of the group did you find helpful? What would you change?
  b. *Probe*:

5. How has your involvement in the peer support groups impacted your recovery process?

Probe: Did you receive useful information?

Probe: Did you feel emotionally supported by the other participants or facilitators?

Probe: Did you feel that the group provided concrete strategies to help deal with challenges in your recovery?

Probe: Did your participation make you feel more connected to the OHSU programme broadly?

6. Have you made any lifestyle changes (*e.g., nutrition, stress management, sleep, weight control and physical activity*) because of your participation in the groups?
7. What do you think are the benefits of participating in a peer support group? What do you think are the potential harms of participating?
8. What was the important factor that allowed you to feel comfortable participating in this group?
9. What were some of the challenges of participating in this group? (e.g., inconvenient time and not enough support).

Reframe: What should we do differently in future offerings of this peer support group?

Reframe: Are there resources or things for us to consider that would have helped you participate more effectively in this peer support group?

10. Can you provide us any insights into which aspects of the group were most effective for people recovering from COVID‐19? Which aspects were least effective?

11. How do you plan to sustain the social or professional relationships that have formed from this group?
12. Do you have any other positive or negative experiences or suggestions that you would like to share?
